# Phase Noise Compensation Algorithm for Space-Borne Azimuth Multi-Channel SAR

**DOI:** 10.3390/s24144494

**Published:** 2024-07-11

**Authors:** Lu Bai, Wei Xu, Pingping Huang, Weixian Tan, Yaolong Qi, Yuejuan Chen, Zhiqi Gao

**Affiliations:** 1 College of Information Engineering, Inner Mongolia University of Technology, Hohhot 010051, China; 20221800100@imut.edu.cn (L.B.); hwangpp@imut.edu.cn (P.H.); wxtan@imut.edu.cn (W.T.); qiyaolong@imut.edu.cn (Y.Q.); chen_yj@imut.edu.cn (Y.C.); gzqnd@163.com (Z.G.); 2 Inner Mongolia Key Laboratory of Radar Technology and Application, Hohhot 010051, China

**Keywords:** space-borne SAR, azimuth multi-channel, Doppler aliasing, phase noise compensation, subspace orthogonal

## Abstract

Azimuth multi-channel synthetic aperture radar (SAR) has always been an important technical means to achieve high-resolution wide-swath (HRWS) SAR imaging. However, in the space-borne azimuth multi-channel SAR system, random phase noise will be produced during the operation of each channel receiver. The phase noise of each channel is superimposed on the SAR echo signal of the corresponding channel, which will cause the phase imbalance between the channels and lead to the generation of false targets. In view of the above problems, this paper proposes a random phase noise compensation method for space-borne azimuth multi-channel SAR. This method performs feature decomposition by calculating the covariance matrix of the echo signal and converts the random phase noise estimation into the optimal solution of the cost function. Considering that the phase noise in the receiver has frequency-dependent and time-varying characteristics, this method calculates the phase noise estimation value corresponding to each range-frequency point in the range direction and obtains the phase noise estimation value by expectation in the azimuth direction. The proposed random phase noise compensation method can suppress false targets well and make the radar present a well-focused SAR image. Finally, the usefulness of the suggested method is verified by simulation experiments.

## 1. Introduction

Because of its all-day, all-weather, strong penetration and high-quality imaging capabilities, the space-borne azimuth multi-channel SAR has been widely used in many key fields such as disaster measurement, resource exploration, marine surveying, and mapping. The space-borne azimuth multi-channel SAR system can effectively solve the conflict between azimuth resolution and range swath, and it effects high-resolution wide-swath (HRWS) imaging. It has become the most mainstream high-resolution wide-swath SAR imaging system both domestically and internationally [1,2,3,4]. Scholars have provided it a great deal of attention and have researched it extensively. However, the pulse repetition frequency (PRF) of the multi-channel SAR system is less than the real Doppler bandwidth, the Doppler spectrum of the received echo in each channel of the azimuth multi-channel SAR system is aliasing in the base band frequency range, and the Doppler spectrum is usually multiple aliasing. Therefore, separating the unambiguous spectrum from the aliasing Doppler spectrum, (that is, Doppler ambiguity resolution), is a core issue in azimuth multi-channel SAR imaging [5].

The space-borne multi-channel system requires precise knowledge of the relative locations of the channels, as well as constant channel properties for optimal deblurring. However, for the actual space-borne multi-channel SAR system, due to the influence of processing technology, temperature, radiation, environment, and equipment, there are inevitably amplitude and phase errors between the receiving channels. These non-ideal factors will affect the deblurring performance of the space-borne multi-channel SAR system, resulting in false targets in the SAR image. Therefore, it is necessary to study the phase error estimation and calibration method of space-borne multi-channel SAR [6,7,8,9]. In 2016, Guo Xiaojiang proposed an improved channel-error-calibration method, which transforms the Doppler variable covariance matrix into a constant covariance matrix, reducing the amount of calculation and improving the estimation accuracy [10]. In 2019, a channel phase error estimation method based on the weighted back-projection algorithm was proposed. This method maximizes the image intensity by gradient descent method to estimate the channel phase error, which is suitable for multi-channel SAR imaging [11]. In 2021, a phase-compensation method combining network construction (NC) and phase gradient autofocus (PGA) is proposed [12]. In the same year, a new channel error calibration algorithm based on orthogonal projection theory was studied. The algorithm does not require matrix eigenvalue decomposition, which avoids signal leakage under a low signal-to-noise ratio [13]. In 2022, a method for estimating the phase error between channels using the minimum norm in the image domain was proposed. This method can obtain higher estimation accuracy by using the focused high signal-to-noise ratio (SNR) image [14]. In 2023, Zhao Xingjie et al. proposed a new algorithm for estimating the unbalanced phase of the channel and further proposed a channel calibration scheme [15]. In the same year, a space-borne multi-channel SAR imaging algorithm was proposed. The algorithm can solve the problem of false targets in the image caused by channel imbalance caused by target motion and is suitable for the presence of multiple maritime moving targets [16]. In 2024, a phase-error-calibration method based on the Least Spectrum Difference (LSD) is proposed. This method performs operations in the Range-Doppler domain, saving a lot of computational costs [17].

The research on the amplitude and phase error of space-borne multi-channel SAR systems has been relatively mature, but for multi-channel SAR systems, the influence of phase noise generated by channel receivers on SAR signal processing is easily ignored. In the existing research, the phase error between channels is generally regarded as a fixed error, but the random phase noise generated by the receiver obeys uniform distribution and has range frequency dependence and azimuth time dependence; that is, the phase noise corresponding to each sampling point in a channel is different. In this paper, the imaging geometry model and echo characteristics of space-borne azimuth multi-channel SAR system are analyzed first. Then the influence of random phase noise on the echo spectrum and pulse compression results of space-borne azimuth multi-channel SAR are studied. Random phase noise causes false targets in pulse compression and imaging results. Finally, in order to suppress false targets, according to the orthogonality between the signal subspace and the noise subspace [18,19,20], this paper proposes a phase noise compensation method for space-borne azimuth multi-channel SAR. The method first transforms the acquired SAR two-dimensional time domain data into the frequency domain, constructs the covariance matrix of each frequency point signal according to the two-dimensional frequency domain data, and obtains the signal subspace and noise subspace matrix through feature decomposition. Since the signal subspace and the noise subspace are orthogonal, a cost function can be constructed. The random phase noise estimation is obtained by solving the function. Based on the frequency-dependent characteristics of phase noise, the phase noise of each range frequency point of the SAR echo data is calculated separately. Based on the time-varying characteristics of phase noise, the mean value of phase noise of all azimuth sampling points corresponding to each distance frequency point is taken as the phase noise estimation value of the frequency point. The random phase noise produced by each channel receiver’s operation can be effectively compensated for by the suggested method so that the echo signal after the compensation of the phase noise can present an accurate, focused SAR image.

The following is the main chapter structure of this article. In the second chapter, the imaging principle of the space-borne azimuth multi-channel SAR system is introduced, and the multi-channel SAR echo signal model is established. In the third chapter, the effects of Doppler aliasing and phase noise on the spectrum and pulse compression results of space-borne multi-channel SAR signals are analyzed. In the fourth chapter, a phase noise compensation method for space-borne azimuth multi-channel SAR is proposed to compensate for the channel mismatch caused by random phase noise and suppress the generation of false targets. In the fifth chapter, the effectiveness of the method is verified by MATLAB simulation experiments.

## 2. Space-Borne Azimuth Multi-Channel SAR Imaging Principle

### 2.1. Imaging Geometry Model

The space-borne azimuth multi-channel SAR system sets multiple linearly arranged receiving channels along the radar course, and multiple receiving channels receive radar echoes at a low-pulse repetition frequency at the same time, which increases the number of spatial samples in a pulse repetition interval (PRI) and compensates for the lack of time-sampling rate [1]. It not only avoids the range ambiguity but also improves the azimuth resolution. The space-borne SAR azimuth multi-channel system has become the development trend and research hotspot in the sphere of space-borne SAR because of its higher resolution, wider coverage, and stronger anti-interference ability.

**Figure 1:**
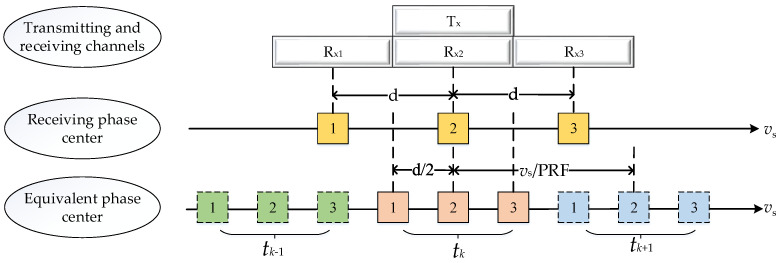
Space-borne azimuth three-channel SAR equivalent phase center.

As shown in Figure 1, the top is a transmitting channel and three receiving channels of the space-borne azimuth multi-channel SAR system. The following is the real receiving phase center of the three receiving channels. Next is the equivalent receiving phase center of the three receiving channels. It is assumed that the space-borne azimuth multi-channel SAR system has a transmitting channel $T_{x}$ for transmitting linear frequency modulation signals. After these linear frequency modulation signals reach the ground and are reflected by ground objects, some linear frequency modulation signals with ground object information will be received by the receiving channel of the SAR system. The three receiving channels are linearly arranged in the azimuth direction, which are $R_{x1}$, $R_{x2}$, and $R_{x3}$, respectively. The receiving phase center of each receiving channel is the geometric midpoint of the channel, which is represented by a yellow box in Figure 1. The serial number in the box represents the serial number of the receiving channel. The center distance between two adjacent receiving channels is d. The azimuth multi-channel SAR system is transmitted by the transmitting channel, and the echo signals received by different channels can be equivalent to the echo signals transmitted and received by the same virtual channel, which is called the equivalent phase center (EPC). The position of EPC is at the midpoint between the transmitting phase center and the receiving phase center. The midpoint of the yellow boxes 1 and 2 is the equivalent phase center of the receiving channel 1; that is, the pink box 1. Therefore, the equivalent phase center spacing is 2/d. Figure 1 shows the equivalent phase centers in three adjacent pulse times $t_{k-1}$, $t_{k}$, and $t_{k+1}$, and different pulse times are distinguished by different colors. In a pulse repetition time, the moving distance of the antenna is $v_{s}/\mathrm{PRF}$.

**Figure 2:**
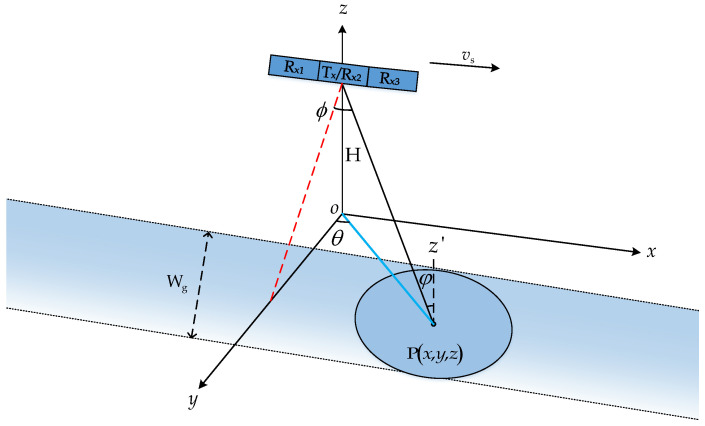
Imaging geometry model of space-borne azimuth three-channel SAR.

The three-dimensional imaging geometry model of the space-borne azimuth multi-channel SAR system (taking three channels as an example, K = 3) is shown in Figure 2. The *xoy* coordinate axis is established on the ground, and the positive half-axis of the *z*-axis is perpendicular to the ground. *z*’ is parallel to the *z*-axis. The height of the space-borne SAR system is H, and it sails forward along the *x*-axis at a speed of $v_{s}$. The lower rectangular bar is the radar beam coverage during the navigation process. W_g_ represents the mapping bandwidth, and the elliptical area is the coverage area of the unit time radar. Supposing that there is a point target P in the coverage area, the position of the point P on the coordinate axis is (x,y,z). The line between SAR and point target P is the slant range vector, and the red dotted line is the shortest slant range from SAR to point target P. The blue solid line is the mapping of the slant vector on the *xoy* plane. The incident angle φ of the point target is the angle between the radar’s slant range vector and *z*’. The cone angle ϕ is the angle formed by the slant range vector and the shortest slant range, and azimuth angle θ is the angle formed by the mapping of the slant range vector on the xoy plane and the *y*-axis.

### 2.2. Echo Signal Model

It is assumed that the space-borne SAR azimuth multi-channel system has one transmitting channel and odd K receiving channels in the azimuth direction. The range and azimuth time are represented by t and η, respectively. The (K+1)/2 channel, which is the intermediate channel, is used as the reference channel. Supposing that the azimuth time η is 0, the position of the reference channel is (0,0,0). The two-dimensional time-domain echo signal received by the reference channel after being reflected by the ground object is indicated as:

(1) $$s_{0}\left(t,\eta \right)=∭\sigma \left(x,y,z\right)\omega_{a}\left(\eta -\frac{x}{v_{s}}\right)\omega_{r}\left(t-\frac{R_{\mathrm{total},0}\left(x,y,z,\eta \right)}{c}\right)\exp\left(-j2\pi \frac{R_{\mathrm{total},0}\left(x,y,z,\eta \right)}{\lambda }\right)dxdydz$$

where, $\sigma \left(x,y,z\right)$ is the backscattering coefficient of the point target P, $\omega_{a}$ is the azimuth two-way beam pattern, $\omega_{r}$ is the pulse envelope, and $R_{\mathrm{total},0}\left(x,y,z,\eta \right)$ symbolizes the two-way distance from the radar reference channel to the ground target point when the reference channel is transmitted and received.

Since the height H value of space-borne SAR is very large, in the geometric relationship, $y^{2}+{\left(H-z\right)}^{2}\gg x^{2}$, and $y^{2}+{\left(H-z\right)}^{2}\gg {\left(x-v_{s}\eta \right)}^{2}$, $R_{\mathrm{total},0}\left(x,y,z,\eta \right)$ can be approximated as follows:

(2) $$\begin{array}{ll} R_{\mathrm{total},0}\left(x,y,z,\eta \right) & =\sqrt{x^{2}+y^{2}+{\left(H-z\right)}^{2}}+\sqrt{{\left(x-v_{s}\eta \right)}^{2}+y^{2}+{\left(H-z\right)}^{2}}\\ & \approx \sqrt{y^{2}+{\left(H-z\right)}^{2}}+\frac{x^{2}}{2\sqrt{y^{2}+{\left(H-z\right)}^{2}}}+\sqrt{y^{2}+{\left(H-z\right)}^{2}}+\frac{{\left(x-v_{s}\eta \right)}^{2}}{2\sqrt{y^{2}+{\left(H-z\right)}^{2}}}\\ & =2\sqrt{y^{2}+{\left(H-z\right)}^{2}}+\frac{x^{2}+{\left(x-v_{s}\eta \right)}^{2}}{2\sqrt{y^{2}+{\left(H-z\right)}^{2}}}\\ & =2\sqrt{y^{2}+{\left(H-z\right)}^{2}}+\frac{x^{2}+{v_{s}}^{2}{\left(\left(x/v_{s}\right)-\eta \right)}^{2}}{2\sqrt{y^{2}+{\left(H-z\right)}^{2}}} \end{array}$$

When the azimuth time is η, the position of the *k*th channel is $\left(x_{k},0,0\right)$, k=1,2,⋯,K. The echo signal of the *k*th channel is expressed as:

(3) $$s_{k}\left(t,\eta \right)=∭\sigma \left(x,y,z\right)\omega_{a}\left(\eta +\frac{\Delta x_{k}}{v_{s}}\right)\omega_{r}\left(t-\frac{R_{\mathrm{total},k}\left(x,y,z,\eta \right)}{c}\right)\exp\left(-j2\pi \frac{R_{\mathrm{total},k}\left(x,y,z,\eta \right)}{\lambda }\right)dxdydz$$

where,

(4) $$\Delta x_{k}=x_{k}-x=\left(\frac{K+1}{2}-k\right)\cdot d$$

Since $y^{2}+{\left(H-z\right)}^{2}\gg x^{2}$ and $y^{2}+{\left(H-z\right)}^{2}\gg {\left(x-v_{s}\eta \right)}^{2}$, $R_{\mathrm{total},k}\left(x,y,z,\eta \right)$ can be approximated by:

(5) $$\begin{array}{ll} R_{\mathrm{total},k}\left(x,y,z,\eta \right) & =\sqrt{x^{2}+y^{2}+{\left(H-z\right)}^{2}}+\sqrt{{\left(x-v_{s}\eta +\Delta x_{k}\right)}^{2}+y^{2}+{\left(H-z\right)}^{2}}\\ & \approx \sqrt{y^{2}+{\left(H-z\right)}^{2}}+\frac{x^{2}}{2\sqrt{y^{2}+{\left(H-z\right)}^{2}}}+\sqrt{y^{2}+{\left(H-z\right)}^{2}}+\frac{{\left(x-v_{s}\eta +\Delta x_{k}\right)}^{2}}{2\sqrt{y^{2}+{\left(H-z\right)}^{2}}}\\ & =2\sqrt{y^{2}+{\left(H-z\right)}^{2}}+\frac{x^{2}+{\left(x-v_{s}\eta +\Delta x_{k}\right)}^{2}}{2\sqrt{y^{2}+{\left(H-z\right)}^{2}}}\\ & =2\sqrt{y^{2}+{\left(H-z\right)}^{2}}+\frac{x^{2}+{v_{s}}^{2}{\left(\left(x/v_{s}\right)-\left(\eta -\left(\Delta x_{k}/v_{s}\right)\right)\right)}^{2}}{2\sqrt{y^{2}+{\left(H-z\right)}^{2}}} \end{array}$$

where the *x*-axis forward is taken as the forward distance, $\Delta x_{k}$ symbolizes the forward distance from the *k*th channel to the reference channel (intermediate channel), and $R_{\mathrm{total},k}\left(x,y,z,\eta \right)$ represents the two-way distance from the ground target point $P\left(x,y,z\right)$ when the reference channel of the radar is transmitted and the *k*th channel is received.

From Formulas (1)–(4), it can be seen that the received signal of channel *k* can be viewed as the azimuth time shift of the reference channel signal, that is:

(6) $$s_{k}\left(t,\eta \right)\approx s_{0}\left(t,\eta -\left(\Delta x_{k}/v_{s}\right)\right)$$

In this paper, lowercase *s* and uppercase *S* are used to distinguish time-domain signals from frequency-domain signals. If the PRF is larger than the Doppler bandwidth, the above equation is converted into a two-dimensional frequency domain to obtain:

(7) $$S_{k}\left(f_{r},f_{a}\right)\approx S_{0}\left(f_{r},f_{a}\right)\exp\left(-j2\pi f_{a}\frac{\Delta x_{k}}{v_{s}}\right)$$

## 3. Doppler Aliasing and Phase Noise Effects

### 3.1. Doppler Aliasing

**Figure 3:**
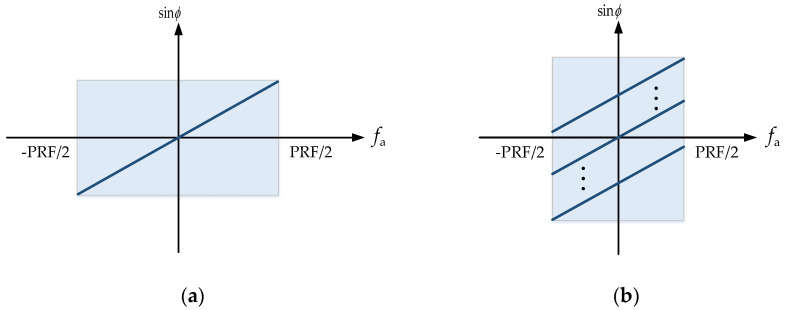
Doppler spectrum of space-borne SAR: (**a**) Doppler spectrum of single-channel system; and (**b**) Doppler spectrum of azimuth multi-channel system.

The PRF of the multi-channel SAR system is generally low. A Doppler frequency point has several echoes that all correspond to the same cone angle ϕ in the Doppler frequency domain. The functional connection between ϕ and the Doppler frequency $f_{a}$ is $f_{a}=\left(2v_{s}\sin\phi \right)/\lambda$. Figure 3 depicts the corresponding connection between $f_{a}$ and sinϕ when the Doppler center is 0 Hz. It can be seen that the PRF of the traditional single-channel system will be greater than the Doppler bandwidth, and the azimuth Doppler spectrum is not blurred, as shown in Figure 3a. The PRF of the multi-channel system is low, and the signal received by each channel is undersampled, which will cause the Doppler spectrum to be blurred, as shown in Figure 3b. Therefore, the unambiguous Doppler spectrum should be restored before the azimuth multi-channel imaging.

In fact, in the space-borne azimuth multi-channel SAR system, the PRF is often smaller than the Doppler bandwidth, and the ratio of the Doppler bandwidth to the PRF is defined as the ambiguity number. Assuming that there are odd N fuzzy numbers, and the fuzzy number is independent of the base-band frequency, considering the random phase noise generated by the receiver, the echo signal received by the kth (k=1,2,⋯,K) channel in the two-dimensional frequency domain is represented as follows:

(8) $$S_{k}\left(f_{r},f_{a}\right)\approx \exp\left(j\varphi_{k}\left(f_{r},f_{a}\right)\right)\sum_{l=-\left(N-1\right)/2}^{\left(N-1\right)/2}S_{0}\left(f_{r},f_{a}+l\cdot \mathrm{PRF}\right)\exp\left(-j2\pi \frac{\Delta x_{k}}{v_{s}}\cdot \left(f_{a}+l\cdot \mathrm{PRF}\right)\right)+N_{k}\left(f_{r},f_{a}\right)$$

where,

(9) $$-\mathrm{PRF}/2\leq f_{a}\leq \mathrm{PRF}/2$$

(10) $$l=-\left(N-1\right)/2,-\left(N-3\right)/2,\ldots ,\left(N-1\right)/2$$

where $f_{r}$ is the range frequency and $f_{a}$ is the azimuth frequency. $\varphi_{k}\left(f_{r},f_{a}\right)$ is the random phase error generated by the receiver in the *k*th channel. Therefore, $\varphi_{k}\left(f_{r},f_{a}\right)$ is a random variable with range frequency dependence and azimuth time dependence. N⋅PRF represents the effective bandwidth of the main lobe of the two-way antenna. In order to effectively suppress orientation blur, the number of azimuth-receiving channels must be greater than the number of blurs. $S_{0}\left(f_{r},f_{a}+l\cdot \mathrm{PRF}\right)$ is an unambiguous SAR echo signal envelope with a frequency shift of l⋅PRF. l is the frequency shift multiple of PRF, and $\Delta x_{k}$ is the distance between the kth-receiving channel and the transmitting channel. $N_{k}\left(f_{r},f_{a}\right)$ is Gaussian white noise.

The two-dimensional frequency domain signals of K channels are represented in matrix form as follows:

(11) $$S{\left(f_{r},f_{a}\right)}_{\left[K\times 1\right]}=\Gamma Q\left(f_{a}\right)S_{0}\left(f_{r},f_{a}\right)+n$$

where,

(12) $$S{\left(f_{r},f_{a}\right)}_{\left[K\times 1\right]}={\left[S_{1}\left(f_{r},f_{a}\right),S_{2}\left(f_{r},f_{a}\right),\ldots ,S_{K}\left(f_{r},f_{a}\right)\right]}^{T}$$

(13) $$\Gamma_{\left[K\times K\right]}=\mathrm{diag}\left[e^{j\varphi_{1}},e^{j\varphi_{2}},\ldots ,e^{j\varphi_{K}}\right]$$

(14) $$\varphi_{1}=E\left[\varphi_{1}{\left(f_{r},f_{a}\right)}_{\left[Nr\times Na\right]}\right],\varphi_{2}=E\left[\varphi_{2}{\left(f_{r},f_{a}\right)}_{\left[Nr\times Na\right]}\right],\cdots ,\varphi_{K}=E\left[\varphi_{K}{\left(f_{r},f_{a}\right)}_{\left[Nr\times Na\right]}\right]$$

(15) $$q_{l}{\left(f_{a}\right)}_{\left[K\times 1\right]}={\left[e^{-j2\pi \left(\Delta x_{1}/v_{s}\right)\cdot \left(f_{a}+l\cdot \mathrm{PRF}\right)},\ldots ,e^{-j2\pi \left(\Delta x_{K}/v_{s}\right)\cdot \left(f_{a}+l\cdot \mathrm{PRF}\right)}\right]}^{T}$$

(16) $$Q{\left(f_{a}\right)}_{\left[K\times N\right]}=\left[q_{-\left(N-1\right)/2}\left(f_{a}\right),\ldots ,q_{\left(N-1\right)/2}\left(f_{a}\right)\right]$$

(17) $$S_{0}{\left(f_{r},f_{a}\right)}_{\left[N\times 1\right]}={\left[S_{0}\left(f_{r},f_{a}-\left(\left(N-1\right)/2\right)\cdot \mathrm{PRF}\right),\ldots ,S_{0}\left(f_{r},f_{a}+\left(\left(N-1\right)/2\right)\cdot \mathrm{PRF}\right)\right]}^{T}$$

(18) $$n_{\left[K\times 1\right]}={\left(N_{1}\left(f_{r},f_{a}\right),N_{2}\left(f_{r},f_{a}\right),\ldots ,N_{K}\left(f_{r},f_{a}\right)\right)}^{T}$$

where $\mathrm{diag}\left[\cdot \right]$ denotes vector diagonalization and ${\left[\cdot \right]}^{T}$ denotes matrix transpose. $q_{l}\left(f_{a}\right)$ is the steering vector, which indicates the response of all array elements of the array antenna to the narrowband source with the unit energy. $Q\left(f_{a}\right)$ represents the steering vector matrix composed of steering vectors from N directions. $S{\left(f_{r},f_{a}\right)}_{\left[K\times 1\right]}$ represents that $S\left(f_{r},f_{a}\right)$ is a matrix of K rows and 1 columns, and $\left[K\times 1\right]$ is the dimension information of $S\left(f_{r},f_{a}\right)$. $\varphi_{1}{\left(f_{r},f_{a}\right)}_{\left[Nr\times Na\right]}$ represents the phase noise matrix of the first channel, which has $N_{r}$ rows and $N_{a}$ columns. $N_{r}$ represents the number of sampling points in the range direction, and $N_{a}$ represents the number of sampling points in the azimuth direction.

### 3.2. Phase-Noise Characteristics

In practical space-borne SAR applications, due to the instability of the transmitter frequency of each channel, the hardware difference of the receiver, and the different working conditions, there are often harmful random phase noises in the SAR echo. And there is a big difference in the random phase noise of each channel.

**Figure 4:**
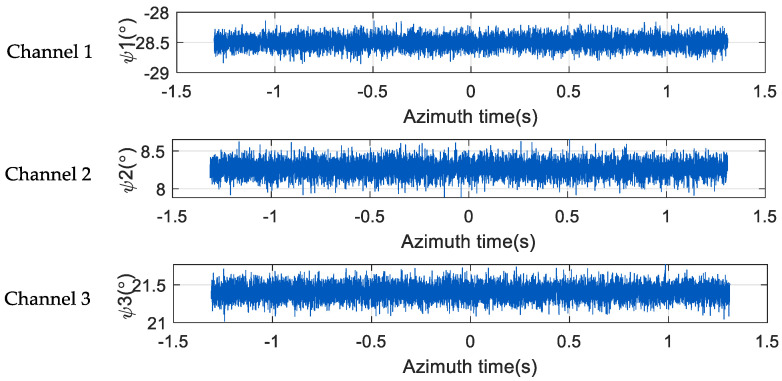
Azimuth time-domain phase diagram of three-channel SAR system.

Taking the space-borne SAR three-channel system as an example, Figure 4 is the relationship between the azimuth time and the angle phase noise of the three channels corresponding to a certain range-frequency point. ψ1, ψ2, and ψ3 are the phase noises of the three channels, respectively, and the phase noises of the three channels are quite different. In each channel, the magnitude of phase noise will fluctuate slightly with the change in azimuth time. That is, in the same channel, the phase noise corresponding to each azimuth sampling point is different, and the phase noise in the receiver has time-varying characteristics in the azimuth direction.

**Figure 5:**
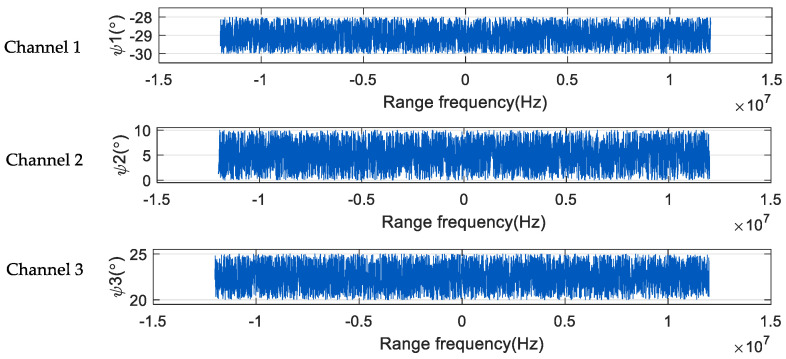
Range-frequency-domain diagram of phase of three-channel SAR system.

Figure 5 shows the variation of the phase noise of the three channels with the range frequency. It can be seen from the figure that in each channel, the phase noise changes with the change of the distance frequency, and the change range is large. The phase noise of the three channels obeys uniform distribution. The value range of ψ1 is (−30°, −28°), the value range of ψ2 is (0°, 10°), and the value range of ψ3 is (20°, 25°). In the same channel, the phase noise corresponding to each range frequency point is different; that is, the phase noise in the receiver has frequency-dependent characteristics in the range direction.

**Figure 6:**
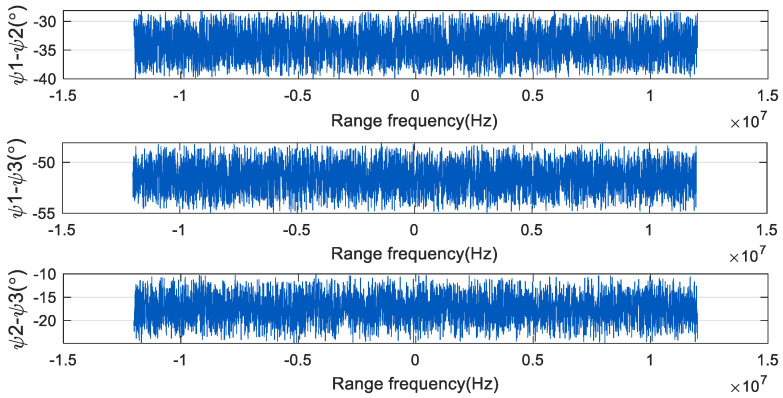
The range frequency domain diagram of the phase difference of each channel in the three-channel SAR system.

The phase noise of each two channels in Figure 5 is subtracted to obtain the difference of the phase noise of each channel, as shown in Figure 6. The phase noise in the three channels is different, so there is a large phase difference between the phases of each channel. This affects the subsequent signal-processing process, resulting in false targets in pulse compression and imaging results.

### 3.3. Phase Noise Effect

The existence of these phase noises will increase the complexity of signal processing, resulting in false targets in the imaging results, which seriously affects the quality of SAR images and the accuracy of subsequent target recognition. The space-borne azimuth three-channel SAR system with random phase noise is simulated and verified below. The second channel in the middle is taken as the reference channel. It is presumed that there is no phase noise in the second channel, and there is a uniform distribution of phase noise in the first and third channel receivers. The system simulation data are shown in Table 1.

**Figure 7:**
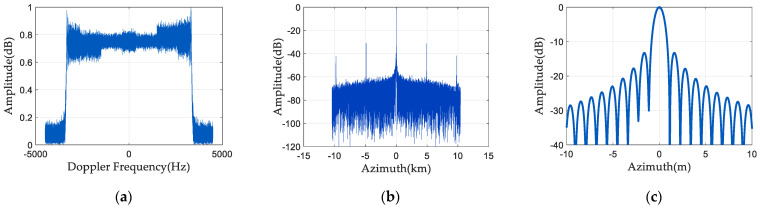
Spectrum and pulse compression results 1 before phase noise compensation: (**a**) signal frequency spectrum; (**b**) pulse compression result; and (**c**) enlarged pulse compression result.

**Figure 8:**
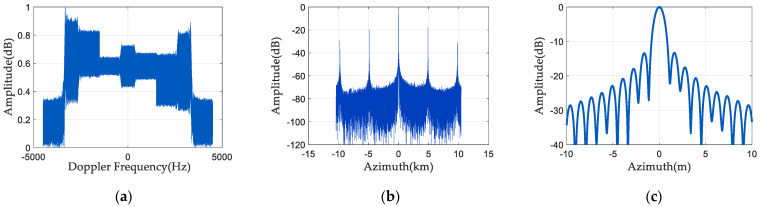
Spectrum and pulse compression results 2 before phase noise compensation: (**a**) signal frequency spectrum; (**b**) pulse compression result; and (**c**) enlarged pulse compression result.

Figure 7 and Figure 8 are the simulation results of spectrum and pulse compression after adding phase noise. As Figure 7 illustrates, (0°, 10°), (−10°, 0°) uniformly distributed phase noise is supplied to the first and third channels, respectively. As shown in Figure 8, (−22°, −18°), (18°, 22°) uniformly distributed phase noise is added to the first and third channels, respectively. Figure 7a and Figure 8a are the spectrograms in the presence of random phase noise, and Figure 7b and Figure 8b are the pulse compression results in the presence of random phase noise. Figure 7c and Figure 8c are the enlarged images of the pulse compression results in the presence of random phase noise. It can be seen from Figure 7 and Figure 8 that the presence of random phase noise will degrade the spectrum quality of the signal and cause false targets in the pulse compression results. Therefore, it is necessary to study a phase noise compensation method for space-borne azimuth multi-channel SAR.

## 4. Phase Noise Compensation Method

### 4.1. Phase Noise Compensation Idea

Assuming that the number of channels in the space-borne azimuth multi-channel SAR system is K, the azimuth sampling point is $N_{a}$, and the range sampling point is $N_{r}$, then the radar echo data received by the *k*th $\left(k=1,2,\ldots ,K\right)$ channel are a matrix of $N_{r}\times N_{a}$, represented by $ss_{k}\left(t,\eta \right)$. In the actual working process of the space-borne SAR azimuth multi-channel system, due to the influence of processing technology, environment, temperature, and receiver working state, each channel receiver will produce a different random phase noise, which is manifested as frequency dependence in range direction and time dependence in the azimuth direction. The phase noise of the kth channel is represented by $\varphi_{k}\left(t,\eta \right)$, which is also the matrix of $N_{r}\times N_{a}$. Therefore, the echo signal with random phase noise overlaid is the echo data that are sent into the next signal processing step; that is, $ss_{k}\left(t,\eta \right)=\overset{\wedge }{ss_{k}}\left(t,\eta \right)\exp\left(j\varphi_{k}\left(t,\eta \right)\right)$, where $\overset{\wedge }{ss_{k}}\left(t,\eta \right)$ is the original radar echo data without random phase noise. This paper’s primary goal is to compensate for the phase noise superimposed on the radar echo signals of each channel so that the radar echo data are closer to the real radar echo data. In this way, the quality of the SAR image after echo reconstruction is further improved, and the emergence of false targets is avoided, so that the subsequent image-based target feature extraction and target recognition operations are more accurate.

In order to express the formula in the proposed method more clearly, we use $ss_{k}\left(t,\eta \right)$ to represent the two-dimensional time-domain signals in both the range and azimuth directions. The signals in the range-frequency domain and the azimuth time domain are represented by $Ss_{k}\left(f_{r},\eta \right)$. The two-dimensional frequency-domain signal with both range- and azimuth-frequency domains is represented by $SS_{k}\left(f_{r},f_{a}\right)$. In this article, all variables are represented in italic letters, and non-variables are represented in orthographic letters. The format of the subscript is the same as above. The vector and matrix are represented by bold italic letters, and the corresponding dimension information is added in the lower right corner.

In this paper, a phase-noise-compensation method for the space-borne azimuth multi-channel SAR is proposed. The number of fuzzy components is used to define the signal and noise subspaces. Based on the orthogonality of the signal and noise subspaces, the cost function is built. The phase-noise approximation corresponding to each sampling point of each channel is obtained by calculating the optimal solution of the cost function. Considering the receiver’s phase noise’s range-frequency dependency, the phase-noise estimation value corresponding to each frequency point is calculated by dividing the received two-dimensional SAR data into frequency points. Considering the azimuth time dependence of the receiver’s phase noise, since the phase noise follows the uniform distribution, the mean value of the phase noise at each sampling point in the azimuth direction is used as the phase-noise estimation value corresponding to the frequency points of each channel.

### 4.2. Phase-Noise-Compensation Steps

1. The acquired 2D time-domain echo signal of each channel of SAR is transformed into the 2D frequency domain;

The two-dimensional time domain data $ss_{k}\left(t,\eta \right)$ of each channel echo signal of the space-borne azimuth multi-channel SAR system are subjected to range Fourier transform to obtain the azimuth time domain and range-frequency-domain data $Ss_{k}\left(f_{r},\eta \right)$ of each channel echo signal.

(19) $$Ss_{k}{\left(f_{r},\eta \right)}_{\left[N_{r}\times N_{a}\right]}=\mathrm{RFFT}\left\{ss_{k}{\left(t,\eta \right)}_{\left[N_{r}\times N_{a}\right]}\right\}$$

where $f_{r}$ is the range frequency, $\mathrm{RFFT}\left\{\;\right\}$ signifies the range Fourier transform, t is the distance time, η is the azimuth time, k=1,2,…,K, K is the number of channels.

To acquire the two-dimensional frequency-domain data $SS_{k}\left(f_{r},f_{a}\right)$ of each channel echo signal, the azimuth Fourier transform is applied to the azimuth time domain and range-frequency-domain data $Ss_{k}\left(f_{r},\eta \right)$.

(20) $$SS_{k}{\left(f_{r},f_{a}\right)}_{\left[N_{r}\times N_{a}\right]}=\mathrm{AFFT}\left\{Ss_{k}{\left(f_{r},\eta \right)}_{\left[N_{r}\times N_{a}\right]}\right\}$$

where $f_{a}$ denotes the azimuth frequency and $\mathrm{AFFT}\left\{\;\right\}$ signifies the azimuth Fourier transform.

2. The covariance matrix is constructed based on the 2D frequency-domain signal and the signal subspace and noise subspace matrix are obtained by feature decomposition.

The 2D frequency-domain echo signal $SS_{k}\left(f_{r},f_{a}\right)$ has $N_{r}$ frequency points in the range direction. Based on the frequency-dependent characteristics of random phase noise in the range direction, the echo signal $SS_{k,n_{r}}{\left(f_{a}\right)}_{\left[1\times N_{a}\right]},\left(n_{r}=1,2,\ldots ,N_{r}\right)$ corresponding to each range frequency point of $SS_{k}\left(f_{r},f_{a}\right)$ is separated and put into the loop, and every frequency-point signal’s related covariance matrix is computed independently. Then, $SS_{k,n_{r}}\left(f_{a}\right)$ is the row vector matrix of 1 row and $N_{a}$ column. The row vector matrices for all K channels’ frequency points are combined to obtain $SS_{n_{r}}\left(f_{a}\right)$:

(21) $$SS_{n_{r}}{\left(f_{a}\right)}_{\left[K\times N_{a}\right]}={\left[SS_{1,n_{r}}{\left(f_{a}\right)}_{\left[1\times N_{a}\right]},SS_{2,n_{r}}{\left(f_{a}\right)}_{\left[1\times N_{a}\right]},\ldots ,SS_{K,n_{r}}{\left(f_{a}\right)}_{\left[1\times N_{a}\right]}\right]}^{T}$$

where ${\left[\;\right]}^{T}$ denotes matrix transpose.

The covariance matrix corresponding to each frequency point signal is expressed as:

(22) $$R_{n_{r}}{\left(f_{a}\right)}_{\left[K\times K\right]}=E\left\{SS_{n_{r}}\left(f_{a}\right)\times SS_{n_{r}}{\left(f_{a}\right)}^{H}\right\}$$

where E represents the mean value. The superscript H signifies the conjugate transpose.

Each frequency signal’s covariance matrix is broken down by feature:

(23) $$R_{n_{r}}{\left(f_{a}\right)}_{\left[K\times K\right]}=U_{n_{r}}\Sigma_{n_{r}}{U_{n_{r}}}^{H}$$

where,

(24) $$\Sigma_{n_{r}\left[K\times K\right]}=\mathrm{diag}\left[\lambda_{1,n_{r}},\lambda_{2,n_{r}},\ldots ,\lambda_{N,n_{r}},\lambda_{(N+1),n_{r}},\ldots ,\lambda_{K,n_{r}}\right]$$

where $\lambda_{1,n_{r}}\geq \lambda_{2,n_{r}}\geq \ldots \geq \lambda_{N,n_{r}}\geq \lambda_{(N+1),n_{r}}=\ldots =\lambda_{K,n_{r}}=\sigma^{2}$, $\sigma^{2}$ is noise power, $\mathrm{diag}\left[\;\right]$ represents vector diagonalization, and $\lambda_{1,n_{r}},\lambda_{2,n_{r}},\ldots ,\lambda_{N,n_{r}}$ represents the power of N signal components corresponding to the $n_{r}th$ frequency point. The eigenvectors corresponding to each eigenvalue can form a signal subspace, denoted by $U_{s,n_{r}}$. $\lambda_{N+1,n_{r}},\ldots ,\lambda_{K,n_{r}}$ represents the power of $\left(K-N-1\right)$ noise components and the eigenvector corresponding to each eigenvalue can form a noise subspace, denoted by $U_{n,n_{r}}$.

3. To determine the phase-noise estimate corresponding to each frequency point of each channel, the cost function is created.

The above noise subspace is obtained by statistics of data with random phase noise, so the noise subspace vector and the steering vector with random phase noise are orthogonal. The estimation of random phase noise at each frequency point can be converted into settling the following optimization issues:

(25) $$\left\{\begin{array}{l} \min\sum_{l=-\left(N-1\right)/2}^{\left(N-1\right)/2}{\left(\Gamma_{n_{r}}\left(f_{a}\right)\cdot q_{l}\left(f_{a}\right)\right)}^{H}U_{n,n_{r}}{U_{n,n_{r}}}^{H}\left(\Gamma_{n_{r}}\left(f_{a}\right)\times q_{l}\left(f_{a}\right)\right)\\ \mathrm{diag}\left[\Gamma_{n_{r}}\left(f_{a}\right)\right]w=1 \end{array}\right.$$

where the phase noise matrix $\Gamma_{n_{r}}\left(f_{a}\right)$ of the $n_{r}th$ frequency point is depicted as:

(26) $$\Gamma_{n_{r}}{\left(f_{a}\right)}_{\left[K\times K\right]}=\mathrm{diag}\left[e^{j\varphi_{1}{,}_{n_{r}}},\ldots ,e^{j\varphi_{k}{,}_{n_{r}}},\ldots ,e^{j\varphi_{K,n_{r}}}\right]$$

(27) $$\varphi_{1,n_{r}}=E\left[\varphi_{1,n_{r}}{\left(f_{a}\right)}_{\left[1\times Na\right]}\right],\ldots ,\varphi_{k,n_{r}}=E\left[\varphi_{k,n_{r}}{\left(f_{a}\right)}_{\left[1\times Na\right]}\right],\ldots ,\varphi_{K,n_{r}}=E\left[\varphi_{K,n_{r}}{\left(f_{a}\right)}_{\left[1\times Na\right]}\right]$$

where $\varphi_{k,n_{r}}$ is the mean value of phase noise of $N_{a}$ sampling points in the echo signal corresponding to the *k*th channel and the $n_{r}th$ frequency point. $q_{l}\left(f_{a}\right)$ is the steering vector. $w={\left[0,0,\ldots ,1,\ldots 0,0\right]}_{K\times 1}^{T}$ represents the middle channel as the reference channel.

The Lagrange multiplier method is used to estimate the phase noise. Therefore, at the $n_{r}th$ frequency point, the estimated value of the phase noise corresponding to each azimuth sampling point is:

(28) $$\Gamma_{n_{r}}{\left(f_{a}\right)}_{\left[K\times K\right]}=\mathrm{diag}\left[\frac{\Omega^{-1}w}{w^{H}\Omega^{-1}w}\right]$$

where,

(29) $$\Omega_{\left[K\times K\right]}=\sum_{l=-\left(N-1\right)/2}^{\left(N-1\right)/2}\mathrm{diag}{\left[q_{l}\left(f_{a}\right)\right]}^{H}U_{n}{U_{n}}^{H}\mathrm{diag}\left[q_{l}\left(f_{a}\right)\right]$$

This paper considers that the phase noise generated by the receiver is uniformly distributed random phase noise. By computing the mean value of the phase noise of each sampling point in the azimuth direction, the random phase noise $\overset{\wedge }{\Gamma_{n_{r}}}$, corresponding to each frequency point of each channel, is obtained:

(30) $$\overset{\wedge }{\Gamma_{n_{r}}}=\mathrm{mean}\left(\Gamma_{n_{r}}\left(f_{a}\right)\right)$$

where $\mathrm{mean}\left(\;\right)$ signifies the average value of phase noise at all sampling points in the channel. $\overset{\wedge }{\Gamma_{n_{r}}}$ is not a variable, so it is represented by non-bold positive parameters.

4. Random phase noise compensation.

The random phase noise $\overset{\wedge }{\Gamma_{n_{r}}}$ corresponding to all frequency points of each channel is constructed as a random phase-noise matrix:

(31) $$\overset{\wedge }{\Gamma }={\left[\begin{array}{ccc} \overset{\wedge }{\Gamma_{1}} & \cdots & \overset{\wedge }{\Gamma_{1}}\\ \vdots & \cdots & \vdots \\ \overset{\wedge }{\Gamma_{n_{r}}} & \cdots & \overset{\wedge }{\Gamma_{n_{r}}}\\ \vdots & \cdots & \vdots \\ \overset{\wedge }{\Gamma_{N_{r}}} & \cdots & \overset{\wedge }{\Gamma_{N_{r}}} \end{array}\right]}_{N_{r}\times N_{a}}$$

According to the assessed value of phase noise, phase-noise compensation is performed on the received echo signal:

(32) $$\overset{\wedge }{SS_{k}\left(f_{r},f_{a}\right)}=SS_{k}\left(f_{r},f_{a}\right)\cdot \overset{\wedge }{\Gamma }$$

Finally, the signal after phase noise compensation is reconstituted and imaged to gain the focused SAR image.

### 4.3. Phase Noise Compensation Algorithm Flow

**Figure 9:**
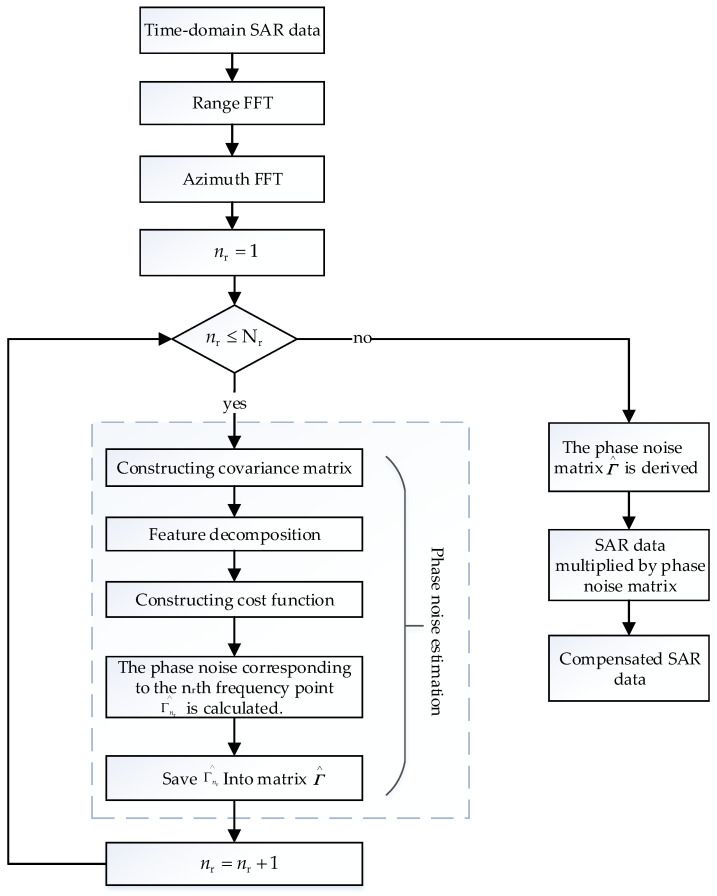
Phase-noise-compensation algorithm flow.

The complete process of the random phase-noise compensation method for the space-borne azimuth multi-channel SAR is demonstrated in Figure 9. Firstly, the SAR two-dimensional time-domain echo matrix is subjected to the range Fourier transform to obtain the echo matrix in the azimuth time domain and the range frequency domain. Then, the azimuth Fourier transform is conducted to obtain the two-dimensional frequency domain echo matrix. $n_{r}$ represents the distance frequency sequence, and there is a total of $N_{r}$ distance frequency points. Firstly, $n_{r}$ is assigned to 1, $N_{r}$ range frequency points are traversed by cyclic judgment, and the phase noise estimation value of the echo signal corresponding to each range frequency point is calculated, respectively. The specific phase-noise-estimation process is as follows: The noise subspace is acquired by constructing the covariance matrix for eigenvalue decomposition. The cost function is structured in accordance with the orthogonality of signal and noise subspaces, and the estimator of random phase noise is transformed into the optimization problem of solving the cost function. The random phase noise estimation value corresponding to the $n_{r}th$ frequency point is calculated and stored in the random phase noise matrix $N_{r}$. Through the loop, the final derived $N_{r}$ contains $N_{r}\times N_{a}$ phase noise estimates. Finally, the SAR data are multiplied by the random phase-noise matrix to compensate for the random phase noise of the SAR signal. By reconstructing and imaging the compensated multi-channel SAR signal, a clearly focused SAR image can be obtained.

### 4.4. Comparison of Computational Complexity

It is assumed that the space-borne azimuth multi-channel SAR system has K receiving channels. The sampling points of the original data in the range and azimuth directions are $N_{r}$ and $N_{a}$, respectively. The SAR echo data received by each channel are an $N_{r}\times N_{a}$ matrix. First, the original data are subjected to distance FFT, which requires $N_{a}N_{r}/2\times \log_{2}\left(N_{r}\right)$ times of complex multiplication. Then, the azimuth FFT is performed, which requires $N_{a}N_{r}/2\times \log_{2}\left(N_{a}\right)$ times complex multiplication. Then, the phase-noise estimates of each frequency point of SAR data are calculated separately by using the cycle. There are a total of $N_{r}$ frequency points in the distance direction, so it is necessary to perform $N_{r}+1$ times of loop judgment and $N_{r}+1$ times of assignment operation. In the specific phase-noise-estimation process, solving the covariance matrix requires $K\times N_{a}$ times multiplication and $K\left(N_{a}-1\right)$ times addition. Feature decomposition requires $K^{2}$ times multiplication and $K^{2}$ times addition. The cost function is constructed to solve the problem, and the optimal solution of $N_{a}$ times is needed for $N_{a}$ azimuth sampling points. In each process of solving the optimal solution, the calculation of the minimum value requires $N\left(2K^{2}+2K-N-1\right)$ times multiplication and $N\left(2K^{2}-N-3\right)+N$ times addition. The calculation of constraint conditions requires $K^{2}$ times multiplication and $K\left(K-1\right)$ times addition. Then, the mean value of $N_{a}$ optimal solutions is obtained. In the process, $\left(N_{a}-1\right)$ additions and 1 division are needed. Finally, the phase-noise compensation of SAR data is conducted, and $N_{r}\times N_{a}$ multiplication is needed.

Compared with the existing methods, the proposed method considers the range-frequency-dependent characteristics of phase noise in the receiver, so the phase-noise estimates corresponding to each frequency point are calculated, respectively. The number of loop-judgment, assignment-operation, and phase-noise estimation calculations is increased. However, after calculating the frequency points separately, the calculation process of the phase noise estimation value of each frequency point is obviously simplified. Although the number of calculations increases, the calculation speed is faster. This paper also considers the azimuth time-varying characteristics of phase noise, so the mean value of the phase noise estimates of all azimuth sampling points corresponding to a certain frequency point is taken as the phase noise estimate of the frequency point. The calculation of the mean does not increase much computational complexity, but it makes the phase-noise estimation more accurate.

## 5. Simulation Verification

### 5.1. Simulation Environment Settings

- Hardware environment

Simulation instrument—high-performance computer, the CPU is Intel (R) Core (TM) i5-8250U, and the GPU is Intel (R) UHD Graphics 620.

- Software selection

Simulation software—MATLAB R2019b.

- Phase-noise setting

The function “unifrnd (A, B)” in MATLAB software is used to generate a random number sequence that obeys uniform distribution, and it is superimposed on the echo signal of each channel as random phase noise. The left and right endpoints of uniformly distributed random numbers are denoted by A and B, respectively. Here represents the minimum degree and the maximum degree of the uniformly distributed phase noise. The second channel in the middle is used as the reference channel. Phase noise in the first and third channel receivers is considered to be uniformly distributed, whereas there is no phase noise in the second channel. Firstly, it is assumed that the phase-noise distribution in the first and third channels has symmetrical intervals, and the efficiency of the phase compensation technique suggested in this study is examined. Then, the phase-noise distribution of the first and third channels is changed to verify the feasibility of the phase-noise-compensation approach suggested in this study when the phase noise of each channel is irrelevant.

Taking the space-borne azimuth three-channel SAR system as an example, it is simulated and confirmed that the suggested phase noise compensation approach works. Each channel’s phase noise is adjusted using the phase-noise-compensation approach described in this work. Table 1 displays the system simulation data.

### 5.2. One-Dimensional Point Target Simulation

**Figure 10:**
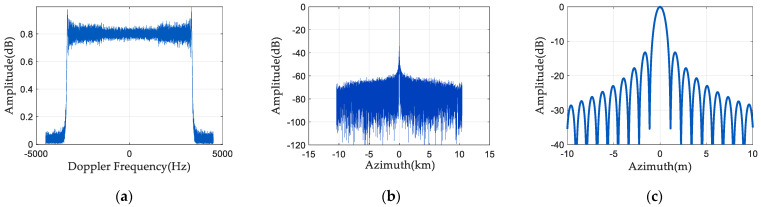
Spectrum and pulse compression results 1 after phase noise compensation: (**a**) signal frequency spectrum; (**b**) pulse compression result; and (**c**) enlarged pulse compression result.

Figure 10 illustrates the spectrum and pulse compression results after phase estimation and compensation using the spaceborne azimuth multi-channel SAR phase-noise-compensation method described in this article. The first channel has (0°, 10°) uniformly distributed phase noise, and the third channel has (−10°, 0°) uniformly distributed phase noise. As Figure 10a illustrates, a better spectrum can be obtained by phase-noise compensation. From Figure 10b,c, it can be seen that the phase-noise-compensation method suppresses the generation of false targets well.

**Figure 11:**
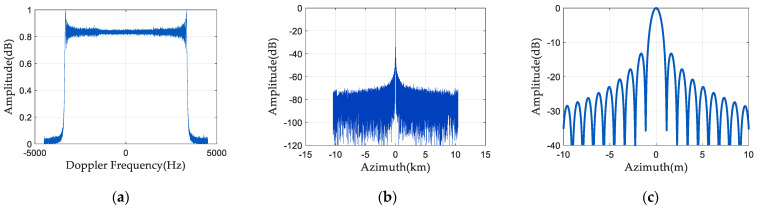
Spectrum and pulse compression results 2 after phase noise compensation: (**a**) signal frequency spectrum; (**b**) pulse compression result; and (**c**) enlarged pulse compression result.

Change the first and third channels’ phase noise distributions, and then do a set of simulation experiments. The first channel adds (−22°, −18°) uniformly distributed phase noise, and the third channel adds (18°, 22°) uniformly distributed phase noise. Figure 11 displays the spectrum and pulse compression results of the echo signal after phase-noise compensation. As seen in Figure 11a, the spectrum is almost the same as the signal spectrum without phase noise. It can be seen from Figure 11b,c that the pulse compression amplitude of the SAR echo signal is also low, and there is no false target.

### 5.3. Two-Dimensional Point Target Simulation

**Figure 12:**
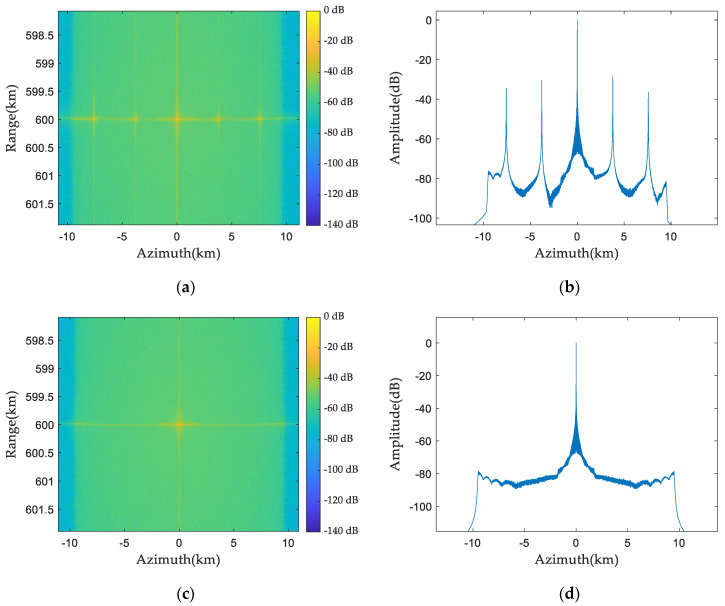
Two-dimensional imaging results of point target before and after phase-noise compensation: (**a**) two-dimensional imaging results before compensation; (**b**) azimuth profile before compensation; (**c**) two-dimensional imaging results after compensation; and (**d**) azimuth profile after compensation.

The following is a two-dimensional simulation experiment of the spaceborne azimuth multi-channel SAR system. It is assumed that the first channel has (−22°, −18°) uniformly distributed phase noise and the third channel has (18°, 22°) uniformly distributed phase noise. Figure 12 is the point targets’ two-dimensional imaging simulation results before and after phase-noise compensation. Figure 12a illustrates the two-dimensional imaging results before phase-noise compensation. There are two false target points, except the point target. Figure 12b is the azimuth profile before phase-noise compensation, in which four false targets appear. Figure 12c is the two-dimensional imaging result after phase-noise compensation, and the point target is rightly focused. It can be seen from Figure 12d that there is no false target in the azimuth profile after phase-noise compensation.

### 5.4. Comparison with Existing Methods

At present, a common phase-error estimation method is the channel-error estimation method based on subspace. The improved phase-error estimation method proposed in [5] obtains the information of the fuzzy component by the spatial-spectrum estimation method, then determines the signal subspace and the noise subspace according to the number of fuzzy components and finally uses the orthogonality of these two subspaces to estimate the channel error. However, the frequency-varying characteristics and time-varying characteristics of the error are not considered. This paper compares the method with the method in [5].

**Figure 13:**
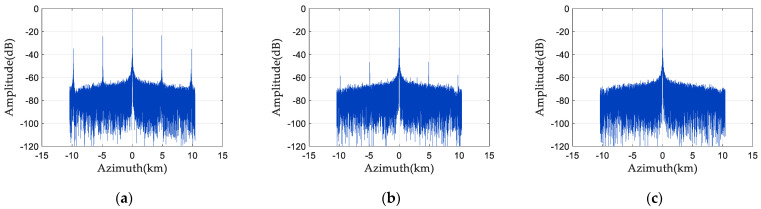
The pulse compression results of the existing method and the suggested method after phase noise compensation: (**a**) before compensation; (**b**) existing method; and (**c**) the suggested method.

Taking the space-borne azimuth three-channel SAR system as the simulation object. In order to confirm if the suggested approach is universal, two completely different sets of phase noise are added to the first and third channels to simulate the random phase noise generated by the receiver. Phase noise uniformly distributed between (−20°, −10°) is introduced into the first channel, while the second channel serves as the reference channel devoid of phase noise. Additionally, the third channel experiences uniformly distributed phase noise ranging between (5°, 8°). As Figure 13 illustrates, the existing method and the method suggested in this article are used to compensate for the phase noise of the SAR echo signal.

As illustrated in Figure 13a, before the phase noise in each channel is compensated, two obvious false targets appear in the pulse compression outcomes due to the channel mismatch, and the pulse compression amplitude of the false target is as high as −30 dB-to-−40 dB. The existing methods usually regard the phase error between the channels of the space-borne azimuth multi-channel SAR system as a fixed value. In Figure 13b, the current method is used for phase-noise compensation. The method is compensated by multiplying the fixed phase noise, and the amplitude of the false target is significantly reduced, which is about −50 dB-to-−60 dB. In contrast, as depicted in Figure 13c, the proposed approach considers both the frequency dependence and time dependence of random phase noise. After phase-noise compensation, there is no false target.

In order to fully prove the effectiveness of the proposed method, we simulate the pulse compression results of phase noise compensation using the existing method and the proposed method under four different phase-noise data. Table 2 is the pulse compression amplitude value of the false target under four sets of phase noise data. Channel 1: (−20°, −10°) indicates that there is a uniform distribution of phase noise from −20° to −10° in Channel 1. Before phase-noise compensation, there are serious false targets in the pulse compression results, and the amplitude is about −20 dB-to-−40 dB. Using the existing method for phase-noise compensation can reduce the false target amplitude by about 20 dB, but the false target is still obvious. The phase-noise compensation method proposed in this paper can well suppress the generation of false targets. The pulse compression amplitude at the corresponding position is below −65 dB. Therefore, the suggested method has a better suppression effect on false targets and is more conducive to subsequent SAR signal processing.

### 5.5. Distributed Target Simulation

**Figure 14:**
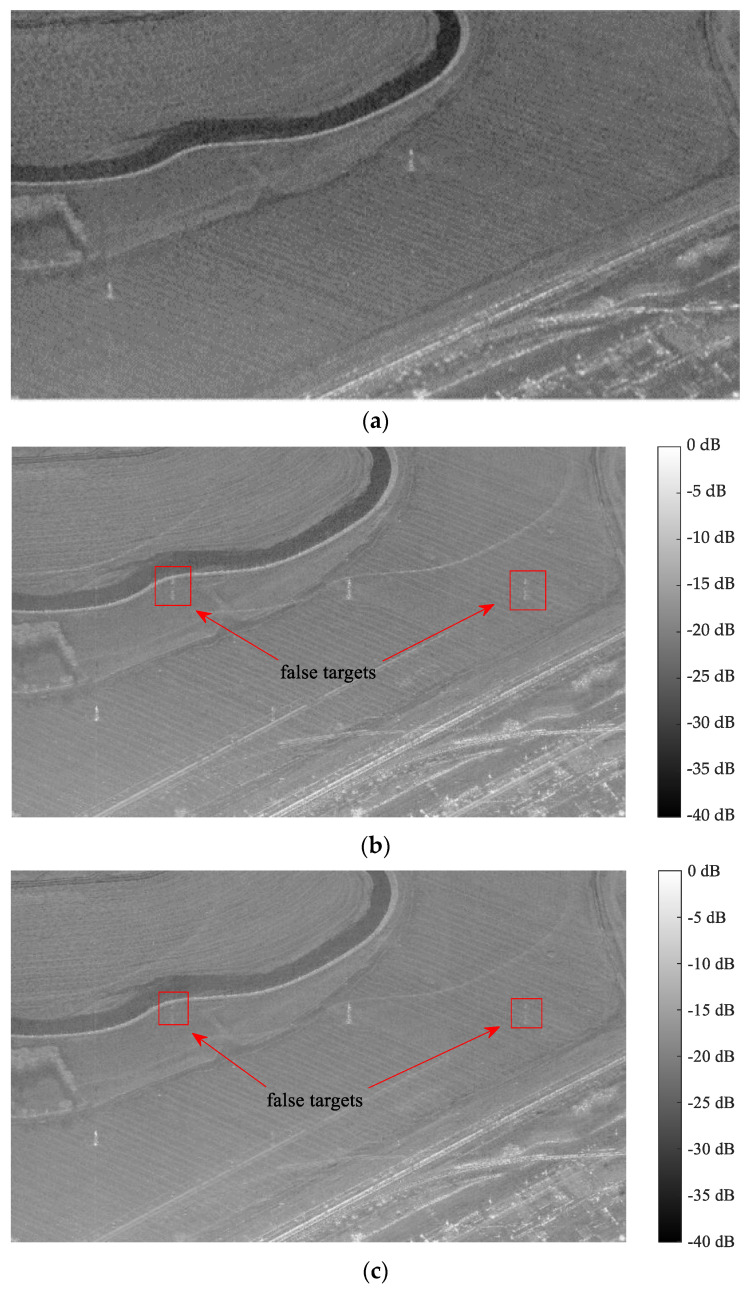

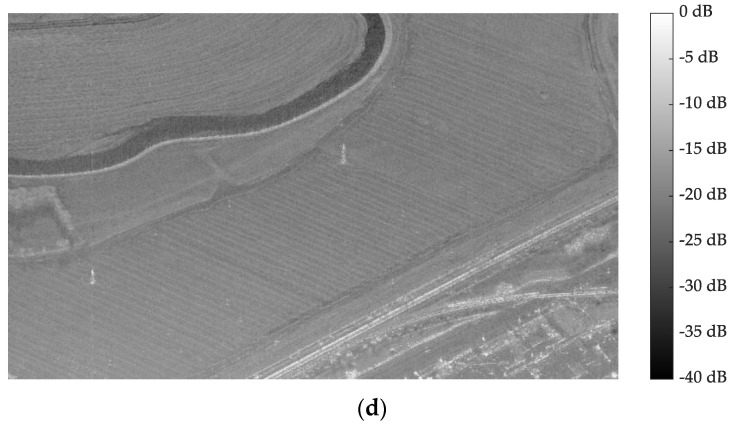
Imaging results of distributed targets before and after phase-noise compensation: (**a**) original SAR image for simulation; (**b**) imaging result before phase-noise compensation; (**c**) the results of phase-noise compensation using existing methods; (**d**) the results of phase-noise compensation using the proposed method.

To further validate the efficacy of the suggested phase-noise-compensation method, the simulation experiment of distributed targets is conducted in this paper. Figure 14 shows the imaging results of distributed targets before and after phase-noise compensation.

Figure 14a is the original SAR image for simulation, and there are two strong targets―lighthouses in the image. Figure 14b is the simulation result of distributed target imaging without compensating the phase noise in the receiver. The quality of SAR images is significantly reduced, the image resolution is low, and false targets appear in the imaging results. Figure 14c shows the imaging results of distributed targets after phase-noise compensation using existing methods. Figure 14d is the imaging result of the distributed target after phase-noise compensation using the method proposed in this paper. It can be seen that the existing method for phase-noise compensation can suppress the false target to a large extent, but there are still weak false targets in the imaging results of distributed targets. However, the phase-noise-compensation method proposed in this paper can completely suppress the generation of false targets, making the SAR image clearer and more focused.

## 6. Conclusions

Space-borne azimuth multi-channel synthetic aperture radar can effectively settle the conflict between azimuth resolution and range swath and achieve high resolution and wide swath imaging. The echo signal received by each receiving channel of the space-borne azimuth multi-channel SAR system obtains an equivalent single-channel signal through digital beamforming technology; that is, the unambiguous spectrum is separated from the aliasing Doppler spectrum. In order to obtain a better deblurring effect, the characteristics of each channel are required to be consistent. However, for the space-borne azimuth multi-channel SAR system, the internal structure of each receiving channel antenna cannot be completely consistent, and each channel receiver will produce different random phase noise when it works. The existing methods generally regard the phase error between channels as a fixed value, but in practice, the phase noise generated by the receiver is different at every moment. These random phase noises are superimposed on the SAR echo signals of each channel, which will cause channel mismatch and lead to false targets in the imaging results. To mitigate the impact of random phase noise on SAR echo-signal reconstruction and imaging and suppress the generation of false targets, a phase-noise-compensation method for space-borne azimuth multi-channel SAR is proposed in this paper. Based on the frequency-dependent and time-varying characteristics of the phase noise generated by the receiver, this paper proposes to put each range frequency point of the two-dimensional SAR echo matrix into the loop and compute the phase-noise estimation corresponding to each range frequency point. By calculating the covariance matrix of each frequency-point-echo signal, the feature decomposition is conducted, and the random phase-noise assessment is transformed into the optimization problem of solving the cost function. The mean value of the phase noise of all the sampling points in the azimuth direction corresponding to each frequency point is calculated as the random phase noise corresponding to the frequency point. Then, the SAR echo signal is multiplied by the phase-noise estimate for phase compensation. The random phase-noise-compensation by this method can well suppress the occurrence of false targets so that the radar presents a well-focused SAR image. Finally, the simulation experiment is conducted. A one-dimensional simulation of two sets of SAR signals with different phase noises is conducted. Without phase-noise compensation, there are obvious false targets in the pulse compression results of one-dimensional point targets. Using the phase-noise-compensation method proposed in this paper for phase compensation can well suppress the occurrence of false targets. Then, the two-dimensional simulation of point targets is conducted by taking one set of data. It can be seen from the SAR imaging that the point target is well-focused after the phase-noise compensation. Finally, a simulation comparison of distributed targets in a certain area is conducted to further verify the effectiveness of the proposed method.

The research on the phase-noise-compensation method of the space-borne azimuth multi-channel SAR system is of great significance for practical application. It covers many fields such as urban planning, map making, environmental monitoring, military reconnaissance, resource exploration, and so on. It provides powerful data support and technical means for all walks of life. By continuously studying more effective error-compensation methods, the imaging quality and measurement accuracy of SAR can be improved, and the application effect of remote sensing can be improved, which provides support for system-performance evaluation and optimization. More importantly, phase-noise compensation can improve the resolution and target recognition ability of the SAR system, enhance military reconnaissance effects, and ensure border security.

The creation of effective phase-noise-estimation algorithms and techniques for SAR system optimization should be the main goals of future research. Parallel computing, distributed computing, and other technologies can be considered to improve computational efficiency. The real-time feedback mechanism and iterative optimization strategy can be considered to dynamically adjust the estimation parameters of phase noise according to the real-time collected multi-channel SAR data and the resulting feedback so as to continuously optimize and improve the estimation results.

## Figures and Tables

**Table 1:** System simulation parameters.

Parameter	Symbol	Value (Unit)
Carrier frequency	$f_{0}$	9.6 (GHz)
Signal bandwidth	$B_{r}$	20 (MHz)
Doppler bandwidth	$B_{a}$	6245.4 (Hz)
Pulse-repetition frequency	PRF	3100 (Hz)
Number of channels	K	3
Pulse width	$T_{r}$	24 (μs)
Adjacent channel spacing	d	1.65 (m)
Number of azimuth samples	$N_{a}$	15,650
Number of range samples	$N_{r}$	6000

**Table 2:** Pulse compression amplitude value of false targets.

Phase Noise Range	Before Compensation	The Existing Method	The Proposed Method
Channel 1: (−20°, −10°) Channel 3: (5°, 8°)	−24.48 dB	−47.27 dB	−66.89 dB
	−35.02 dB	−58.54 dB	−79.77 dB
Channel 1: (0°, 3°) Channel 3: (−12°, −5°)	−29.98 dB	−44.36 dB	−70.95 dB
	−41.75 dB	−55.36 dB	−73.97 dB
Channel 1: (−32°, −28°) Channel 3: (−30°, −25°)	−19.61 dB	−35.50 dB	−76.38 dB
	−36.00 dB	−46.91 dB	−77.81 dB
Channel 1: (15°, 28°) Channel 3: (−3°, 12°)	−23.45 dB	−36.62 dB	−64.92 dB
	−36.12 dB	−52.12 dB	−77.81 dB

## Data Availability

The original contributions presented in the study are included in the article, further inquiries can be directed to the corresponding author.
